# Conformational fingerprinting of tau variants and strains by Raman spectroscopy†

**DOI:** 10.1039/d1ra00870f

**Published:** 2021-02-26

**Authors:** George Devitt, Anna Crisford, William Rice, Hilary A. Weismiller, Zhanyun Fan, Caitlin Commins, Bradley T. Hyman, Martin Margittai, Sumeet Mahajan, Amrit Mudher

**Affiliations:** School of Biological Sciences, Faculty of Environmental and Life Sciences, University of Southampton Highfield Southampton SO17 1BJ UK G.T.Devitt@soton.ac.uk; School of Chemistry, Faculty of Engineering and Physical Sciences, University of Southampton Highfield Southampton SO17 1BJ UK; Institute for Life Sciences, University of Southampton Highfield Southampton SO17 1BJ UK; Department of Chemistry and Biochemistry, University of Denver 2190 E. Iliff Ave. Denver CO 80208 USA; Department of Neurology, Harvard Medical School, MassGeneral Institute for Neurodegenerative Disease, Massachusetts General Hospital Charlestown MA 02129 USA

## Abstract

Tauopathies are a group of disorders in which the deposition of abnormally folded tau protein accompanies neurodegeneration. The development of methods for detection and classification of pathological changes in protein conformation are desirable for understanding the factors that influence the structural polymorphism of aggregates in tauopathies. We have previously demonstrated the utility of Raman spectroscopy for the characterization and discrimination of different protein aggregates, including tau, based on their unique conformational signatures. Building on this, in the present study, we assess the utility of Raman spectroscopy for characterizing and distinguishing different conformers of the same protein which in the case of tau are unique tau strains generated *in vitro*. We now investigate the impact of aggregation environment, cofactors, post-translational modification and primary sequence on the Raman fingerprint of tau fibrils. Using quantitative conformational fingerprinting and multivariate statistical analysis, we found that the aggregation of tau in different buffer conditions resulted in the formation of distinct fibril strains. Unique spectral markers were identified for tau fibrils generated using heparin or RNA cofactors, as well as for phosphorylated tau. We also determined that the primary sequence of the tau monomer influenced the conformational signature of the resulting tau fibril, including 2N4R, 0N3R, K18 and P301S tau variants. These results highlight the conformational polymorphism of tau fibrils, which is reflected in the wide range of associated neurological disorders. Furthermore, the analyses presented in this study provide a benchmark for the Raman spectroscopic characterization of tau strains, which may shed light on how the aggregation environment, cofactors and post-translational modifications influence tau conformation *in vivo* in future studies.

## Abstract 2

We report that the physiochemical properties of the aggregation environment dictate the conformation of tau strains, which can be characterized and distinguished using Raman spectroscopy.

## Graphical abstract

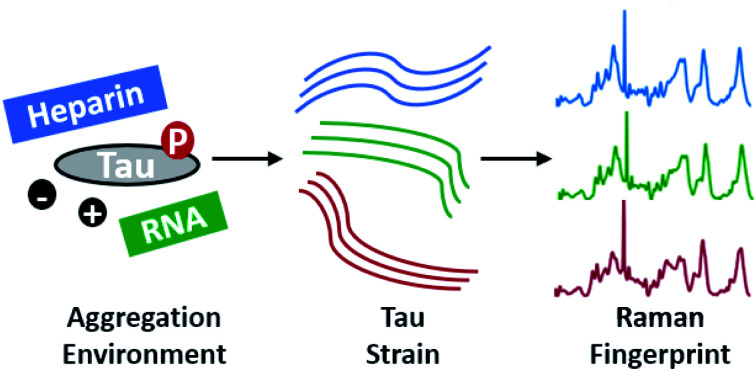

## Introduction

Tauopathies are a group of disorders in which the deposition of abnormally folded tau protein accompanies neurodegeneration. Distinct clinical symptoms and affected neuro-anatomical regions enable disease classification.^1^ The molecular structure of the pathological tau in different tauopathies is variable with respect to several factors including isoform composition, conformation, post translational modification (PTM) pattern, and potentially cofactor incorporation.^2^

Alternative splicing of the microtubule associated protein tau (MAPT) gene results in the generation of six tau isoforms in adult humans, each of which contain microtubule-binding domains with either three or four repeat regions (also known as the repeat-domains). In general, pathological inclusions in Alzheimer's disease (AD) contain fibrils composed of both three-repeat (3R) and four-repeat (4R) tau isoforms, whereas Pick's disease (PiD) fibrils contain 3R isoforms, and progressive supranuclear palsy (PSP) and corticobasal degeneration (CBD) fibrils contain 4R isoforms.^1^ Like other amyloids, these tau fibrils contain an intermolecular cross-β-sheet core composed of amino acids primarily in the repeat-domain.^3^ Structural variations in the loop and turn regions or the inclusion of different amino acids in the individual β-strands of the amyloid core result in distinct conformers/strains of assembled tau protein. Trypsin-resistant amyloid cores of tau fibrils from AD, PiD, CBD and PSP have unique fragment patterns when separated by gel electrophoresis, suggesting that each disease contains a distinct assembled tau conformer/strain.^4^ Atomic-resolution structures of these amyloid cores have been mapped using cryo-electron microscopy (cryo-EM), establishing the existence of distinct conformers of fibrillar tau in AD,^5^ PiD,^6^ CBD^7^ and chronic traumatic encephalopathy (CTE).^8^

The mechanism(s) by which tau aggregates to form fibrils *in vivo* are not known. When aggregation to form tau fibrils is simulated *in vitro*, cofactors are required, possibly encouraging aggregation by overcoming the repulsion between positively charged repeat domains of protein monomers.^9^ Heparin, a polyanionic sulphated glycosaminoglycan (GAG), was first shown to polymerize full-length tau *in vitro*,^10^ whilst other molecules including ribonucleic acid (RNA)^11^ and arachadonic acid^12^ have also been shown to trigger tau polymerization. Cryo-EM has shown that tau fibrils formed from heparin *in vitro* are distinct from those so far seen in human disease.^13^ RNA and heparan sulfate proteoglycans are associated with neurofibrillary tangles of tau in AD,^14–16^ whilst non-proteinaceous densities in the cryo-EM maps of tau fibrils from AD,^5^ PiD,^6^ CTE^8^ and CBD^7^ brain tissue suggest the incorporation of an unknown cofactor.^17^ Ultimately, it remains unknown whether cofactors are a trigger or consequence of tau aggregation *in vivo*.^2^ The role of cofactors in tau aggregation in disease is currently not well established and requires further investigation. Hence, in this study we were motivated to provide proof-of-concept of the possibility that Raman spectroscopy could assist those attempting to decipher the identity and understand the role of cofactors in tau aggregates.

*In vivo*, Tau conformation and assembly can also be directly influenced by post-translational modifications (PTMs). For example, phosphorylation of tau at serine-202, threonine-205 and serine-208, with the absence of phosphorylation at serine-262 leads to spontaneous aggregation of tau *in vitro* in the absence of cofactor.^18^ As well as being conformationally distinct, it has been shown that tau fibrils from AD and CBD have unique patterns of PTMs, suggesting that PTMs may be used as markers to identify tau conformers from different diseases.^19,20^

As alluded to above, the mechanism(s) by which tau aggregates *in vivo* are not known and there is an urgent need to explore the role of factors shown to influence this pathogenic process. The development of methods that can identify tau isoform, conformation, cofactor interaction and PTM may be useful for understanding the interplay between these factors and their role in disease progression. In this study we demonstrate the utility of one such methodology. Raman spectroscopy offers a direct, label-free analysis of vibrational modes within a given sample. These vibrations arise from chemical bonds, enabling unique fingerprinting of different molecules. Chemical bond vibrations are influenced by inter- as well as intra-molecular interactions, the latter in particular, means that Raman spectroscopy is highly sensitive to protein conformation.^21^ While Raman spectroscopy has been shown to provide fingerprints for different amyloid proteins^22^ and fibrillar mutants of α-synuclein,^23^ we have recently shown conformational fingerprinting of Bovine Serum Albumin (BSA), β2-microglobulin (β2M) and tau in their different aggregation states.^21^ Conformational features in terms of secondary and tertiary structures were compositionally disambiguated. The unique conformational fingerprints of monomers, oligomers and fibrils of BSA, β2-microglobulin and tau allowed clear and unambiguous distinction through both direct spectral analysis and unsupervised classification.^24^ Recently Raman spectroscopy was used to identify conformational polymorphism of insulin amyloid fibrils in different buffer conditions.^25^ It is yet to be determined whether fibril polymorphism, aggregation cofactor, PTM or primary sequence have an impact on the Raman spectrum of tau aggregates.

In this study, we have used Raman spectroscopy to characterize the fibril structure of several tau variants and strains generated *in vitro*. We assessed whether the Raman spectra of each fibril population could be distinguished using principal component analysis (PCA) and amide I curve-fitting analysis.^21^ Specifically, we investigated the impact of four principal factors on the Raman fingerprint and tau fibril conformation: (1) aggregation environment (2) cofactor incorporation (3) phosphorylation, and (4) Tau monomer primary sequence. First, we observed that the tau strains that were generated in different aggregation environments had distinct morphologies observed by atomic force microscopy (AFM), as well as distinct secondary structural compositions based on their Raman fingerprints. Next, we identified unique Raman markers for tau fibrils generated in using heparin or RNA cofactors, as well as for phosphorylated tau. Finally, we found that fibrils formed from 0N3R and 2N4R tau isoforms, the P301S–2N4R tau mutant or the K18 tau fragment generated unique Raman fingerprints that enabled their classification. This study highlights the utility of Raman spectroscopy to characterize and distinguish distinct tau fibril populations based on their conformation and unique Raman signatures. These conformational signatures can be used to shed light on the interplay between the aggregation environment, cofactors and post-translational modifications on tau conformation in future studies.

## Results

**Fig. 1:**
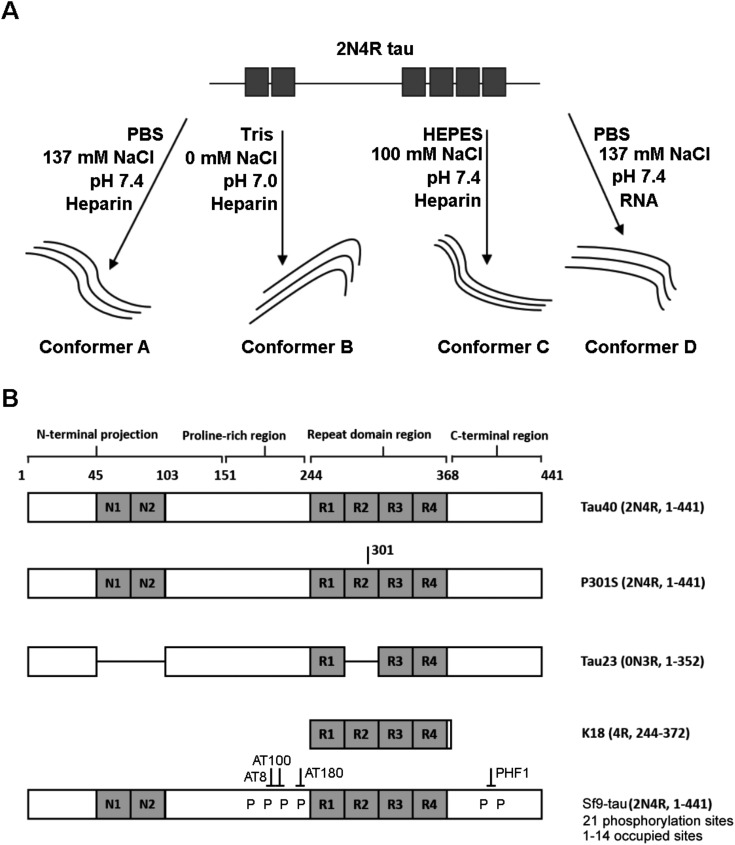
Schematic representing variants and strains used in this work (A) Tau fibril strains were generated in different buffer conditions (*e.g.* PBS, HEPES or Tris) or in the presence a different cofactor (heparin or RNA). (B) Schematic showing primary structure of tau variants used in this work. Fibrils were generated from variants including tau isoforms (tau40 and tau23), the tau mutant P301S, the tau fragment K18 and phosphorylated 2N4R Sf9 tau. Isoform variable regions are shown in grey, including the N-terminal inserts (N1 and N2) and the repeat domain repeats (R1–R4). Tau fibril polymorphism was assessed using Raman spectroscopy.

Fig. 1 is a schematic representation of the proteins and aggregation conditions used in this study. First, distinct fibril strains were generated from recombinant tau monomers *in vitro* using different aggregation environments (Fig. 1A). Next, tau fibrils were generated using heparin or RNA cofactors (Fig. 1A). Finally, fibrils were formed from different variants of monomeric tau in the same aggregation conditions (Fig. 1B). We employed Raman spectroscopy to ascribe conformational signatures to each of the fibril populations formed.

### Different aggregation environments generate distinct tau conformers

We have previously demonstrated that Raman spectroscopy provides unique conformational signatures for fibrils generated from BSA, β2M and tau proteins *in vitro*.^21^ We now wanted to study the impact of different aggregation environments on the conformational signature of aggregate formed, and investigated this for tau fibrils. We incubated 2N4R tau monomers in the presence of heparin at a 2 : 1 molar ratio (protein : heparin) in either PBS buffer (10 mM Na_2_HPO_4_, 2 mM KH_2_PO_4_, 137 mM NaCl, 2.7 mM KCl, 2 mM DTT, pH 7.4) or Tris buffer (25 mM Tris, 2 mM DTT, pH 7.0) in quiescent conditions. These conditions were employed simply to test the impact of one specific environmental factor on conformation when tau's primary structure and aggregating cofactor (heparin) is the same. Additionally we wanted to illustrate the sensitivity of Raman spectroscopy to discriminate between different conformations of otherwise identical proteins.

**Fig. 2:**
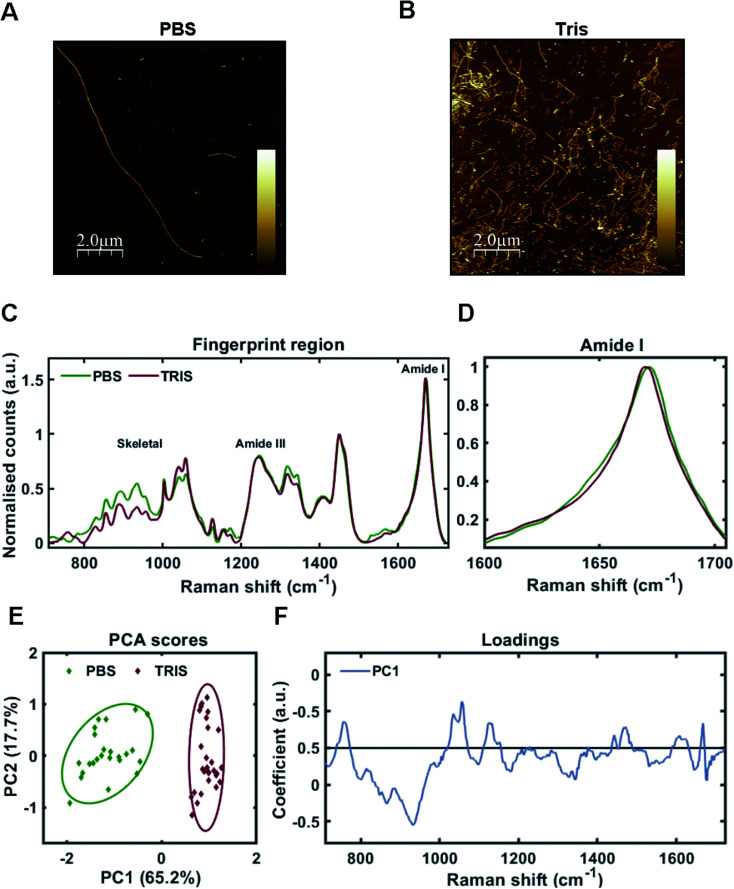
Raman fingerprints of 2N4R-tau fibril strains. (A and B) AFM images of tau fibrils generated from 2N4R tau in PBS buffer using heparin cofactor (A), or in Tris buffer using heparin cofactor (B). Scale bar = 2 μm, *Z* scale = 0 nm–7 nm (A), 0 nm–14 nm (B). (C) Raman spectra of sedimented tau fibrils aggregated in the presence of heparin cofactor generated from 2N4R tau in PBS buffer (green trace), or Tris buffer (red trace). Amide I, amide III and skeletal regions are highlighted. (D) Normalized amide I region for the Raman spectra shown in (C). (E) 2-Dimensional principal component analysis (PCA) scores plot of Raman spectra shown in (C). Each solid diamond represents the PC score of a single spectrum. (F) PC loadings spectra representing the spectral variation responsible for the score across the PC1 axis shown in (E). Raman spectra represent the class means from multiple spectra; PBS-tau fibrils: 24, Tris-tau fibrils: 29.

As one may predict, the two different aggregating environments had a profound influence on the tau conformer generated. AFM revealed that fibrils grown in PBS were a mixture of sparse long fibrils (10 μm+) and short, stubby fibrils (Fig. 2A). Long fibrils were not observed in Tris buffer conditions, with many fibrils found clumped together in high density, suggesting lower stability (Fig. 2B). These observations were reinforced after sedimentation of insoluble tau, with larger pellets observed for tau grown in Tris compared to tau fibrils grown in PBS. This aligns with a previous study using the tau 4R domain fragment.^26^

Raman fingerprints for the tau fibrils grown in each condition are shown in Fig. 2C. β-Sheet conformation was confirmed by the frequency of the amide I band (C=O stretching) ∼1670 cm^−1^ for each strain. We observed a subtle, but repeatable, difference in the amide I peak frequency between each tau strain, with the Tris conformer having an amide I frequency of 1669 cm^−1^ and the PBS conformer having an amide I frequency of 1671 cm^−1^ (Fig. 2D). Importantly, this frequency is sensitive to the number of β-strands in the β-sheet, as well as their orientation, as opposed to the proportion of β-sheet structure, suggesting that each conformer has a different structural architecture.^27^ The amide III frequency (1200–1300 cm^−1^) is sensitive to the dihedral (*Ψ*) peptide bond angles of the protein backbone,^28^ but we observed no clear difference in amide III frequency between each tau strain. Mean Raman spectra may over- or underestimate contributions from individual spectra. Therefore, unsupervised PCA was applied to the Raman spectra in order to classify the data and identify regions of spectral variation between datasets. PCA showed that the individual spectra of each tau strain were distinguished by a single principal component (PC1, Fig. 2E). The loadings showed that the skeletal region (C–C and C–N stretching from 850–1150 cm^−1^) intensity had a large weighting on the PCA scores (Fig. 2F). The skeletal region is conformationally sensitive, but structural assignment is more complex than for the amide I or Amide III bands.^24^

Interestingly, the Raman spectrum for the Tris strain had more intense peaks ∼1050–1070 cm^−1^ in comparison to that for the PBS strain (Fig. 2C and F). These peaks are assigned to sulfate stretches of heparin^29^ (see also further below). This may reflect differential incorporation of heparin in each of the aggregation conditions. As the interaction between tau and heparin is based on electrostatic interactions, the higher salt conditions in the PBS buffer (137 mM NaCl) may have decreased these interactions in comparison to the low salt conditions using Tris buffer (0 mM NaCl). It has been demonstrated that increasing concentrations of NaCl >50 mM decrease heparin-induced tau aggregation kinetics,^26^ whilst 300 mM NaCl prevented the interaction between tau and heparin completely.^30^

These results demonstrate a clear impact of the aggregation environment on the tau conformer formed. To prove that this effect was independent of the cofactor involved in aggregation, we assessed the impact of different aggregation environments on the aggregation of β2M strains, which unlike tau do not require a co factor. Consequently, the Raman spectra of β2M fibrils are less complex than those for tau fibrils. ESI Fig. S1† shows that as was the case for tau, changes to the aggregation environment also influenced the conformers of β2M fibrils that formed. Long-straight and worm-like β2M fibril strains were evident by AFM and they have unique Raman signatures, suggesting distinct β-sheet conformations. This is in agreement with previous studies employing electron paramagnetic resonance (EPR) spectroscopy and Fourier transform infrared spectroscopy (FTIR).^31,32^

It has been demonstrated that tau strains from different tauopathies have cell type specificity that is maintained during pathological transmission, but the specific factors that modulate tau strain conformation and specificity are yet to be determined.^33^ Our data collectively demonstrates that the aggregation environment can have a significant impact on the conformers formed therein irrespective of the cofactor that promotes aggregation. This supports the link between cellular environment and strain conformation.

### Raman fingerprints of tau fibrils are sensitive to different cofactors

**Fig. 3:**
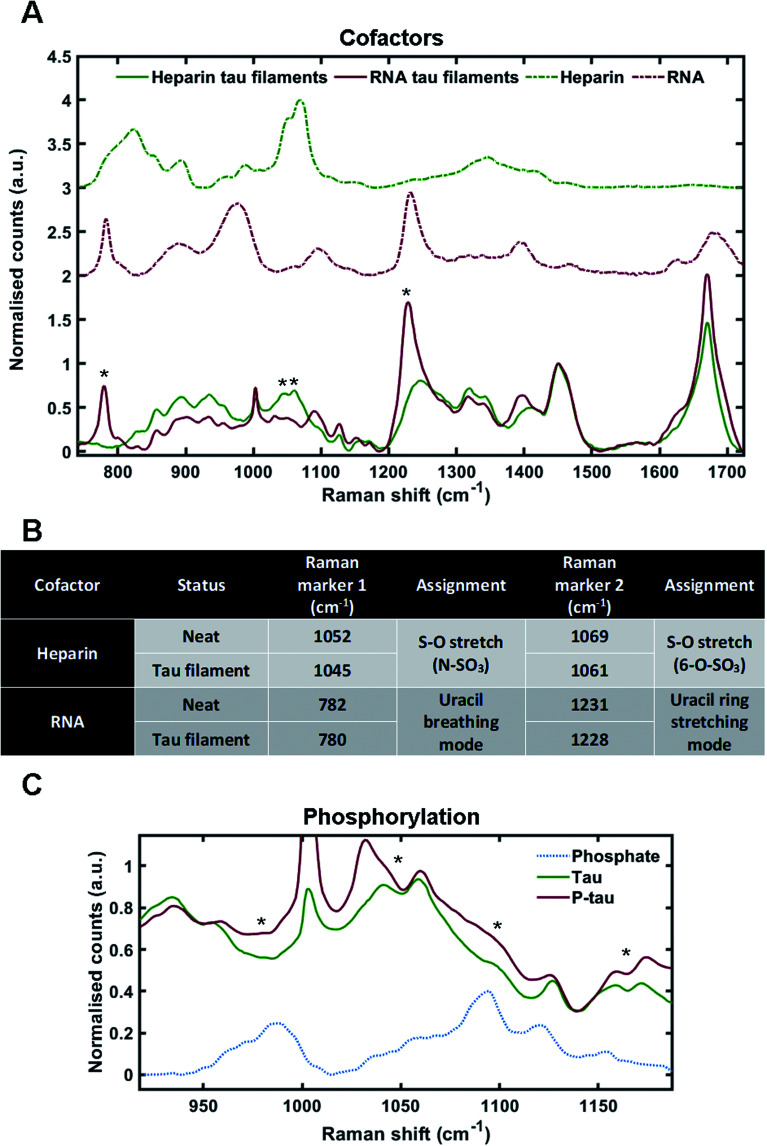
Markers of heparin, RNA and phosphorylation in the Raman spectrum of tau fibrils. (A) Raman spectra of sedimented tau fibrils aggregated in the presence of heparin cofactor (green trace) or RNA cofactor (red trace), as well as pure heparin (dashed green trace) and pure RNA (dashed red trace). Asterixes represent unique markers of cofactors in tau fibril spectra. (B) Table showing the frequency of Raman markers for heparin and RNA cofactors alone (neat) and after sedimentation of tau fibrils. (C) Raman spectra of 2N4R tau fibrils aggregated in the presence of heparin (green, also shown in A), Sf9 2N4R tau aggregated in the presence of heparin (red), and neat sodium phosphate (dashed blue trace). Asterixes represent changes in tau fibril Raman spectrum that align with phosphate peaks from the sodium phosphate spectrum. Raman spectra represent the class means from multiple spectra; heparin–tau fibrils: 24, RNA tau fibrils: 9, heparin: 5, RNA: 3, Phosphorylated tau fibrils: 20, sodium phosphate: 1.

Cofactors are likely to influence tau aggregation in disease so it would be desirable to have a method that enables their identification in the tau aggregates.^17,34^ We therefore investigated whether different cofactors could be identified in the Raman spectrum of tau fibrils. To do this, we incubated tau monomers with polyuridylic acid (Poly(U)) RNA at a molar ratio of 3 : 1 (tau : RNA) – to generate fibrils. Tau fibrils were then isolated from the mixture by sedimentation, washed several times to remove any surface-bound cofactor and then probed by Raman spectroscopy. Tau fibrils generated using heparin (as shown in Fig. 2) were used for comparison. Raman spectra were also acquired of neat heparin and neat RNA (Fig. 3A, green dotted line and red dotted line, respectively). The Raman fingerprints for tau fibrils formed in the presence of either heparin (green line) or RNA (red line) were distinct (Fig. 3A). Unique peaks originating from each cofactor were visible in the Raman fingerprint for each of the tau fibril samples indicated by asterixes (*). In the heparin spectrum, these peaks were assigned to the sulfate S–O stretches at 1052 cm^−1^ (N–SO_3_) and 1069 cm^−1^ (6-O–SO_3_).^29^ In the RNA spectrum, these peaks were assigned to the uracil ring modes at 782 cm^−1^ (ring breathing) and 1231 cm^−1^ (ring stretching).^35^

The incorporation of cofactors into the fibril structure relies on electrostatic interactions between the positively charged tau residues and negatively charged cofactor functional groups, such as the phosphate backbone of RNA or the sulfate groups in heparin.^9^ Raman spectroscopy can be used to study electronic interactions by the observation of frequency shifts in Raman peaks.^36^ Peak shifts for heparin and RNA markers after tau fibril formation are tabulated in Fig. 3B. For heparin, the S–O stretching peaks showed a downshift in frequency in the tau fibril spectrum; 1052–1045 cm^−1^ and 1069–1061 cm^−1^. The uracil ring modes in the RNA spectrum also showed a downshift in the tau fibril spectrum; 782–780 cm^−1^ and 1231–1228 cm^−1^. The larger shifts observed for sulfate stretches in heparin were likely due to direct electronic interaction between the sulfate groups and tau, whereas it is likely that the RNA phosphate backbone interacts with tau and not the RNA uracil ring. Interestingly, the phosphate backbone band ∼900–1000 cm^−1^ was the only region of the RNA spectrum that was not evident in the RNA–tau fibril spectrum, with the tau fibril backbone C–C stretching peaks also observed in this region Fig. 3A. This suggests that the phosphate backbone undergoes a change in structure, likely due to a direct interaction with tau. These findings suggest that the heparin and RNA cofactors were incorporated into the overall fibril structure, in agreement with previous observations.^9,34,37^

### Raman fingerprints of tau fibrils are sensitive to phosphorylation

As cofactors could be detected in the Raman spectrum of tau fibrils, we asked whether it was possible to identify markers of phosphorylation. To do this, recombinant tau was generated in eukaryotic SF9 cells. Mass spectrometry has indicated that tau isolated from SF9 cells contains 21 phosphorylation sites including AD diagnostic AT8, AT100, AT180 and PHF1 sites, and is phosphorylated at 1–14 sites per molecule, as shown in Fig. 1B.^38^ Phosphorylated tau was incubated with heparin at a molar ratio of 2 : 1 (tau : heparin) and the isolated tau fibrils were probed by Raman spectroscopy. Dried sodium phosphate buffer was also probed by Raman spectroscopy in order to assign spectral peaks corresponding to phosphate groups (Fig. 3C). The peak for dibasic phosphate was observed at 987 cm^−1^ (–OPO(3)(2−)) and the peak for monobasic phosphate was observed at 1093 cm^−1^ (–OPO(3)H(−)), in line with previous literature.^39^ In tau fibrils, this region of the spectrum is convoluted due to skeletal C–C/C–N vibrations from tau protein, S–O vibrations from heparin and P–O vibrations from the phosphate groups. Increased intensities in the phosphate vibration spectral region (950–1100 cm^−1^) in the phosphorylated tau fibrils were observed in comparison to non-phosphorylated tau fibrils, aligning with dibasic and monobasic phosphate peaks (Fig. 3C).

### Raman fingerprints of tau fibrils are sensitive to primary sequence

In different tauopathies, different tau isoforms are implicated, for example 4R isoforms aggregate in tauopathies such as PSP, CBD and others,^40^ whilst 3R isoforms aggregate in tauopathies like PiD.^41^ Additionally, in some familial tauopathies point mutations in tau have also been identified.^42^ To investigate the effect of such differences in primary sequence on the Raman fingerprint of tau fibrils, we generated fibrils from the largest tau isoform (2N4R), the shortest naturally occurring isoform (0N3R), and 2N4R tau with a single point mutation (P301S). Additionally, we also used a construct containing only the repeat domain that forms the amyloid core of tau fibrils (K18), as this is heavily implicated in all tau aggregates. The β-sheet secondary structure in tau fibrils is predominantly localized to the repeat domain, whereas the outer regions are less ordered (fuzzy coat). The proportions of these regions differ between 2N4R, 0N3R and K18 tau variants (see Fig. 1B). Therefore, we hypothesized that each variant would have a distinct Raman fingerprint.

**Fig. 4:**
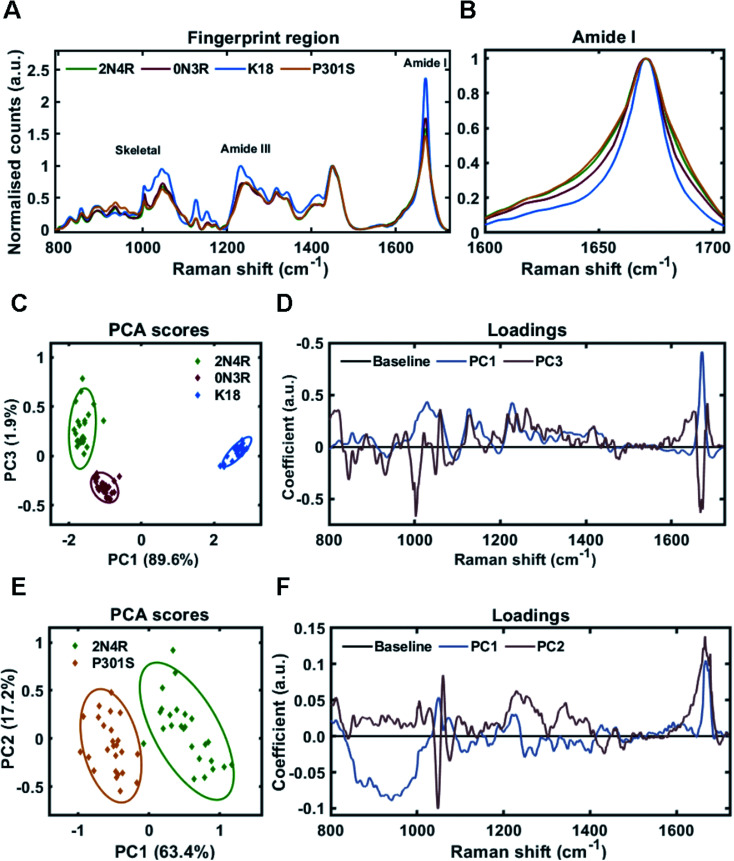
Raman fingerprints of tau fibrils formed from 2N4R, 0N3R, K18 and P301S variants. (A) Raman spectra of sedimented tau fibrils aggregated in HEPES buffer under agitation in the presence of heparin cofactor. Fibrils were generated from the following tau variants; 2N4R tau (green trace), 0N3R tau (red trace), K18 tau (blue trace) and P301S tau (orange trace). Amide I, amide III and skeletal regions are highlighted. (B) Normalized amide I region for the Raman spectra shown in (A). 1600–1705 cm^−1^ shown for clarity. (C) 2-Dimensional principal component analysis (PCA) scores plot of Raman spectra shown in (A) including 2N4R tau (green diamonds), 0N3R tau (red diamonds) and K18 tau (blue diamonds). Each solid diamond represents the PC score of a single spectrum. (D) PC loadings spectra representing the spectral variation responsible for the score across the given PC axis shown in (C). (E) 2-Dimensional principal component analysis (PCA) scores plot of Raman spectra shown in (A) including 2N4R tau (green diamonds) and P301S tau (orange diamonds). Each solid diamond represents the PC score of a single spectrum. (F) PC loadings spectra representing the spectral variation responsible for the score across the given PC axis shown in (E). Raman spectra represent the class means from 25 spectra per class.

Tau fibrils were formed in the presence of heparin at a molar ratio of 2 : 1 (heparin : tau) in HEPES buffer under constant agitation. Fibrils were separated from soluble tau by sedimentation and probed by Raman spectroscopy. The Raman fingerprint of each tau fibril population is shown in Fig. 4A. The Raman fingerprints for 0N3R and 2N4R isoforms were relatively similar, whereas the Raman fingerprint for K18 fibrils was clearly distinct. β-Sheet components in the amide I and amide III region (1670 cm^−1^ and 1233 cm^−1^, respectively) were relatively more intense in the K18 fibril spectrum than for 2N4R and 0N3R fibrils. This suggested that K18 had relatively more β-sheet than 2N4R and 0N3R tau fibrils. This is expected as nonregular/disordered regions outside of the repeat-domain of the protein that make up the ‘fuzzy coat’ are not present in K18 (see Fig. 1B). Similarly, heparin peaks were more intense in the K18 fibril spectrum (∼1045 cm^−1^) as a K18 monomer is smaller than the other isoforms. The frequency of amide I region was identical for each variant, although the width of each amide I band was distinct, suggesting that each fibril variant had a different secondary structural composition (Fig. 4B).

Principal component analysis was performed on the acquired spectra in order to identify spectral variation in an unbiased manner. The scores plot in Fig. 4C shows that 2 principal components (PC1 and PC3) were required to sufficiently distinguish the spectra from 2N4R, 0N3R and K18 fibrils. The loadings for each of these PCs is shown in Fig. 4D. PC1 showed strong positive coefficients for β-sheet components (1672 cm^−1^ – amide I, 1227 cm^−1^ – amide III, 1028 cm^−1^ – skeletal) and heparin (1059 cm^−1^). The PC1 loadings aligned with the scores plot, which showed that K18 fibril spectra had a positive coefficient in comparison to 2N4R and 0N3R fibril spectra, which had negative coefficients. Furthermore, the vibration ∼935 cm^−1^ is very weak in K18 fibrils in comparison to 2N4R and 0N3R fibrils. This vibration was also observed in monomeric tau protein^24^ and was therefore assigned to nonregular structure.

The PC3 axis separates positively scored 2N4R and negatively scored 0N3R fibril spectra. The loadings showed that peaks including amide I β-sheet ∼1669 cm^−1^ and phenylalanine ring breathing mode ∼1002 cm^−1^ were more intense in 0N3R fibril spectra, whereas nonregular and turn structure in the amide I (∼1651 cm^−1^ and ∼1684 cm^−1^) and amide III regions (∼1261 cm^−1^), as well as C–N stretching ∼1128 cm^−1^ were more intense in 2N4R fibril spectra.

As changes in primary sequence had measurable effects on the Raman spectrum, we asked whether a single point mutation (P301S) would result in the formation of fibrils with a distinct conformational fingerprint. The P301S tau mutation occurs within the second repeat of the microtubule binding domain and results in early onset and rapidly progressing Frontotemporal dementia (FTD) with Parkinsonism.^43^Fig. 4A shows that fibrils formed by 2N4R tau with and without the P301S mutation had distinct Raman fingerprints. The spectrum for P301S fibrils had a comparable, but slightly broader amide I region, suggesting a larger ensemble of secondary structures than in the wild type (WT) 2N4R fibrils (Fig. 4B). This was more clearly shown in the amide III region, with P301S fibril amide III spectra centred at 1248 cm^−1^, indicative of nonregular structures, whilst 2N4R fibrils had a more intense peak ∼1236 cm^−1^, indicative of β-sheet structure. These differences were subtle, but consistent, as shown by the PCA scores plot in Fig. 4E. The PCA loadings showed that the skeletal region and the amide I intensity relative to the CH_2_ band played a large role in distinguishing the spectra (Fig. 4F). Importantly, β-sheet related vibrational frequencies at 1667 cm^−1^ (amide I), 1228 cm^−1^ (amide III) were associated more strongly with the spectra of 2N4R fibrils than P301S fibrils.

### Quantitative conformational fingerprinting of tau fibril strains

The Raman amide I band of proteins represents the sum of multiple peaks that each correspond to a different element of secondary structure. Underlying peaks can be resolved by curve-fitting analysis, which enables the assignment of secondary structure. The resulting structural composition can be used as a conformational fingerprint of a given protein/protein ensemble.^24,44–46^ Here, we apply the same curve-fitting method to the amide I spectrum of each fibril strain to establish a unique and quantitative conformational fingerprint for each population of fibrils.

**Fig. 5:**
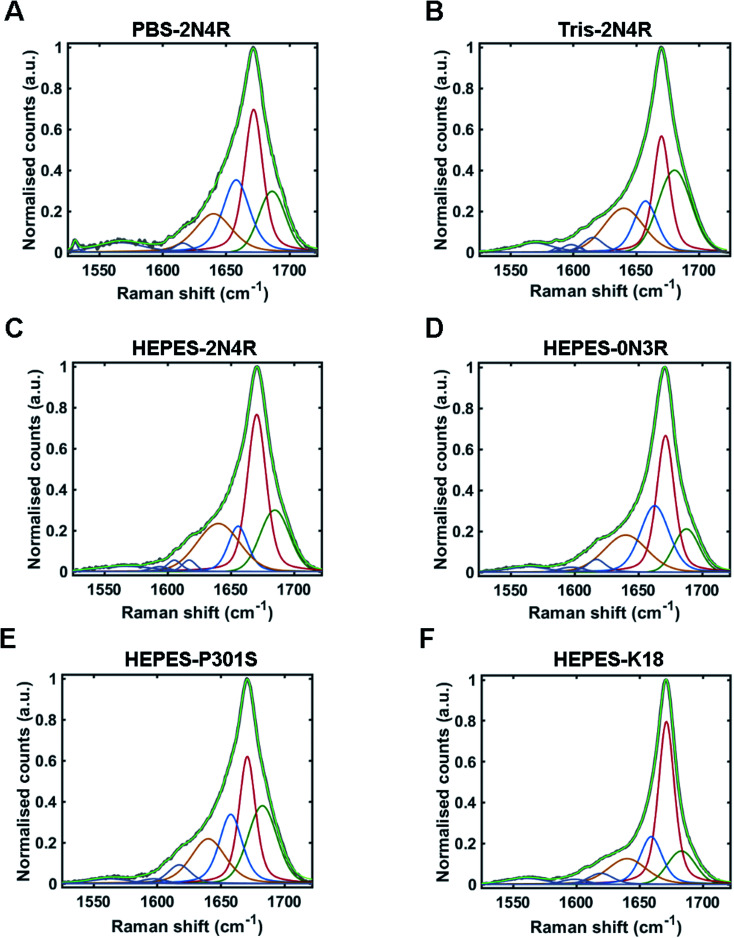
Amide I curve-fitting analysis. Curve-fitting analysis of amide I band (1525–1725 cm^−1^) from tau fibril spectra including; PBS-2N4R (A), Tris-2N4R (B), Hepes-2N4R (C), Hepes-0N3R (D), Hepes-P301S (E) and Hepes-K18 (F). Non-fitted amide I band is shown in grey, with the fitted curve shown in light green. Underlying peaks corresponding to secondary structure are shown in dark green (nonregular), red (β-sheet), blue (turn/helix), and orange (coupling/nonregular). Aromatic amino acid peaks are shown in purple.

We fitted the amide I region between 1525–1725 cm^−1^ using peaks representing aromatic acids (1525–1620 cm^−1^) and secondary structure (1620 cm–1725 cm^−1^). Peaks representing secondary structure were fitted and assigned as follows ∼1655 cm^−1^ (α-helix/turns), ∼1670 cm^−1^ (β-sheet), and ∼1686 cm^−1^ (nonregular). A further peak between 1620–1640 cm^−1^ was also included in the fit. This peak is not well defined and may originate from vibrational coupling and/or nonregular structure.^47–49^ The fitted amide I region for each fibril strain is shown in Fig. 5 and quantified in Table 1. The percentage peak areas were used as a readout of the proportion of secondary structure. Variation in peak widths were also observed. It is well established that the sharpness of the Raman peak is related to structural order, for example in crystals compared to amorphous materials.^50^ Peak width is therefore representative of the distribution of underlying structures and overall conformational order where wider peaks represent a higher distribution of underlying structures or a decrease in structural order.^45^

**Table 1:** Secondary structure composition from amide I curve-fitting analysis

Strain/variant	Nonregular/coupling			Turn/α-helix			β-Sheet			Nonregular		
	Frequency (cm^−1^)	Width (cm^−1^)	Area (%)	Frequency (cm^−1^)	Width (cm^−1^)	Area (%)	Frequency (cm^−1^)	Width (cm^−1^)	Area (%)	Frequency (cm^−1^)	Width (cm^−1^)	Area (%)
PBS-2N4R	1640	35	19	1658	25	26	1672	17	37	1686	25	18
Tris-2N4R	1640	37	22	1657	22	16	1670	16	29	1680	31	33
PBS-2N4R (RNA)	1635	15	5	1657	23	29	1670	16	45	1687	27	21
Hepes-2N4R	1640	39	25	1656	18	12	1670	18	41	1685	27	22
HEPES-0N3R	1640	40	22	1663	27	27	1671	16	37	1688	23	14
HEPES-K18	1640	36	17	1659	21	19	1671	15	50	1683	26	14
HEPES-P301S	1640	32	21	1658	21	22	1671	15	30	1683	27	27

Amide I curve-fitting analysis of tau fibrils formed in PBS or Tris buffer revealed that each strain population had a distinct secondary structural composition. Fibrils formed in Tris contained less β-sheet structure than fibrils formed in PBS (29% and 37%, respectively), less turn/helical structure (16% and 26%, respectively), and more total nonregular/coupling structure (55% and 37%, respectively). In contrast, amide I curve-fitting analysis of β2M fibrils formed in high or low salt conditions revealed that each strain had a comparable secondary structural composition (ESI Fig. S2†), as shown previously by Hiramatsu *et al.* using Fourier-transform infrared spectroscopy (FTIR).^32^ We observed that both strains had 60% total β-sheet structure, although differences in peak frequency (1671 cm^−1^ and 1674 cm^−1^) suggested some difference in β-sheet architecture, as discussed.

Amide I curve-fitting analysis of 2N4R, 0N3R, P301S and K18 tau fibrils generated in HEPES buffer under agitation revealed that each fibrillar variant had a distinct secondary structural composition. 2N4R tau fibrils were composed of 41% β-sheet structure, 12% turn/helical structure and 47% nonregular/undefined structure. As expected, tau fibrils formed from K18 contain the largest proportion of β-sheet structure (50%), as this variant does not contain the nonregular ‘fuzzy coat’ regions. 0N3R tau fibrils contained 37% β-sheet secondary structure and a larger proportion of turns/helices (27%) than 2N4R fibrils (12%).

Interestingly, we observed that tau fibrils with the P301S mutation contained less β-sheet structure (30%) than the WT 2N4R tau fibril population (41%). These findings are in agreement with our observations using PCA, as well as a previous electron paramagnetic resonance (EPR) study that showed increased structural disorder in the fourth repeat and the adjacent C-terminal region of P301S fibrils compared to 2N4R fibrils.^51^

We next performed amide I curve-fitting analysis on the 2N4R tau fibril population formed using RNA cofactor. The Raman spectrum for RNA contained peaks that overlap with the protein amide I band, originating from carbonyl stretching (ESI Fig. S3†) making secondary structural analysis more complex. Therefore, we performed curve-fitting analysis in two ways. First, curve-fitting was performed on the neat RNA carbonyl band and the three resulting peaks were included in the RNA–tau amide I analysis. Second, we performed curve-fitting analysis of the protein amide I band after careful subtraction of the RNA carbonyl spectrum. Each method produced comparable results (ESI Fig. S3†). We noted that heparin had a small peak at 1650 cm^−1^, but subtraction of this peak did not affect our curve-fitting results. We found that the tau fibrils generated in PBS using either heparin or RNA had a distinct secondary structural composition. Fibrils formed using RNA contained more β-sheet structure than those formed with heparin (45% and 37%, respectively) and less nonregular/coupling structure (26% and 37%, respectively). Taken together, these analyses show that Raman spectroscopy can distinguish fibrillar protein strains by their conformational fingerprint using PCA and by their secondary structural composition using amide I curve-fitting analysis.

## Discussion

We have demonstrated that tau fibrils adopt different conformations based on the physiochemical properties of the aggregation environment, the cofactor used for aggregation, the monomer primary sequence, and the presence or absence of disease associated mutations. This validates the utility of Raman spectroscopy for the detection and classification of fibrillar tau variants *in vitro* and provides detailed information about the extraordinary conformational flexibility of the tau molecule. Each of the experimental manipulations noted above generated a unique Raman signature based on its molecular composition and structure. The Raman fingerprint and tau fibril conformation were sensitive to four principal factors; (1) aggregation environment (2) cofactor incorporation (3) PTM, and (4) tau monomer primary sequence. First, we showed that fibrillar tau strains that share an identical primary sequence can be distinguished by Raman spectroscopy based on their distinct conformational signatures, despite the added spectral convolution of a cofactor. Next, we showed that the molecules that are incorporated into the tau fibrils, including heparin and RNA cofactors, as well as PTMs such as phosphorylation, can be identified in the Raman spectrum. Finally, we showed that Raman spectroscopy can be used to classify a range of fibrillar tau variants with different primary sequences including 2N4R, 0N3R, K18 and P301S, each of which was found to have a unique conformational signature. These Raman signatures may serve as vital probes for dissecting the factors *in vitro* that dictate the conformational polymorphism of tau seen in disease. This may shed light on what these factors are *in vivo*, where they must be influencing and possibly even driving aggregation in affected neurons.

### The utility of Raman spectroscopy for characterizing and distinguishing tau conformers

We have previously demonstrated the utility of Raman spectroscopy for distinguishing between aggregates formed from different proteins.^24^ However, given that different conformers of the same protein, in this case tau, are found in different tauopathies^17^ and possibly even in the same tauopathy,^20,52^ it is more important to demonstrate the conformational sensitivity of this methodology for discrimination of different tau conformers beyond distinguishing between monomer, oligomer or fibril of the same tau variant. We have proven this here by showing that fibrils of different tau conformers/variants have unique conformational fingerprints. Fibrils formed *in vitro* from 2N4R and K18 tau have unique amyloid cores, as shown by their unique limited-proteolysis signatures^53^ and differences in backbone mobility.^51^ Furthermore, the amyloid cores from AD and PiD fibrils also extend beyond 4th repeat so it would not be possible for K18 to form these disease conformations *in vitro*. We have demonstrated that the fibrils generated from different tau variants, 2N4R, 0N3R, K18 and P301S, have unique conformational signatures with different proportions of secondary structures as a result of different amino acid compositions. It is such differences in conformation that form the basis of seeding barriers between different amyloid structures.^54^ It has been demonstrated previously that the aggregation of 2N4R tau in the presence of heparin leads to the formation of at least four different conformers of tau fibrils *in vitro* (defined as; snake, twister, hose and jagged).^13^ The conformational signature of tau fibrils observed using Raman spectroscopy in this work represents the average conformation of this fibril population, which includes both the structured (amyloid core) and nonregular regions of tau fibrils (fuzzy coat). We note that the β-sheet content observed in tau fibrils using Raman spectroscopy is higher than expected from cryo-EM analysis.^13^ It has been remarked that amide I curve-fitting overpredicts β-sheet structure in protein fibrils,^55^ possibly due to resonance enhancement as a result of inter-strand coupling of vibrational modes.^56–59^ Regardless, we determined that the P301S mutation caused an increase in nonregular structure in the fibrillar population of tau in comparison to 2N4R WT tau, supporting previous work using EPR.^51^ Importantly, tau conformers formed using a heparin cofactor *in vitro* are likely to be distinct from conformer populations formed in an *in vivo* environment. For example, transgenic P301S tau fibrils from the mouse brain were shown to be far less stable than recombinant P301S tau fibrils formed *in vitro*.^60^

It has been established that unique tau conformers exist in different tauopathies,^4–8,13,61^ and that conformers from disease differ to those formed *in vitro*,^62,63^ possibly due to differences in the available cofactor,^13^ PTM,^19^ local pH or ion environment.^23,25^ Emerging evidence suggests that different populations of soluble tau aggregates may exist between different AD patients and that the presence of a given population may correlate with disease onset and progression.^20^ We have shown that changes in the physiochemical environment can lead to the formation of distinct tau conformers, reinforcing the possibility that different conformers may also occur in different disease settings. In our system, electrostatic interactions with a cofactor also regulates aggregation. Increased charge screening from NaCl in PBS buffer may make tau less conducive to heparin interaction than in low ionic strength Tris buffer. Fibril folding during aggregation may also be influenced by highly kosmotropic phosphate ions and weakly chaotropic chloride ions.^64^ Ion-specific Hofmeister effects can affect amyloidogenic aggregation differently depending on the protein.^65^ Characterizing spectral features of soluble tau aggregates from different disease cases may shed light on what specifically about the pathological aggregated protein correlates with disease progression, although isolating the protein would be necessary for Raman spectroscopy. It is noted that cryo-EM has thus far not identified different conformers from a single tauopathy, for which there may be many possibilities *e.g.* conformers may exist in smaller populations and may not be detected after data ‘averaging’, or a predominant conformer may be established by the end of aggregation and cryo-EM has thus far only been performed with end stage tau fibrils and not on soluble species.

### The utility of Raman spectroscopy for identifying tau cofactors

Molecular structures, including conformations, are a manifestation of the lowest energy or most stable state as a result of the net electronic environment. Raman spectroscopy probes the vibrations of chemical bonds (electrons shared between atoms); while it is sensitive to the presence, quantity, strength and angle of those bonds it is also sensitive to any environmental effects which affect these bonds and their electronic structure.^36,66,67^ Therefore, the addition of any exogenous agent to a tau protein sample will cause changes in the Raman fingerprint provided it effects the electronic environment of the atomic nuclei of the bonds involved. These changes could be due to inter- or intra-molecular interactions and any conformational changes induced by such interactions between tau and the added exogenous agent. We have shown that both RNA and heparin cofactors have unique spectral markers that can be identified in the fingerprints of tau fibrils. Therefore, Raman spectroscopy can be used to detect tau protein and a given cofactor, whilst also providing conformational information. It has been suggested that different cofactors can induce differences in tau fibril conformation, resulting in distinct properties in a cellular environment.^34,68^ We have shown that 2N4R tau fibril populations generated using heparin and RNA cofactors have distinct secondary structural compositions. Both heparin and RNA are polyanionic and interact with tau *via* electrostatic interactions, but differences in the 3-dimensional structure of cofactors may sterically influence specific tau conformations. For example, in this study we used poly(U) RNA, which is a pyrimidine nucleobase, as opposed to bulkier purine nucleobases adenine and guanine. It is possible that pathological tau folding in brains of tauopathy patients, and the resulting conformation of tau fibrils, could be dictated by the cofactors available in the protein's environment. Interestingly, it has been shown that cofactors can cause strain adaptation in the prion protein, with the incorporation of a different cofactor leading to changes in strain conformation and infectivity.^69^ Furthermore, poly(ADP-ribose) has been shown to act as a cofactor for α-synuclein aggregation, leading to the formation of a highly toxic α-synuclein strain.^70^ The aggregated tau conformers found in different tauopathies could each have a unique combination of cofactors depending on their brain region and environment, which may serve as a unique biomarker and therapeutic target. It is possible that the Raman fingerprint of isolated tau conformers may thus provide clues as to the nature of this cofactor, which has thus far been elusive.

### The utility of Raman spectroscopy for identifying tau PTMs

Similarly, we have demonstrated that the phosphorylation of tau produces unique markers in the Raman fingerprint of tau fibrils. These spectral changes are far more subtle than for the incorporation of cofactors, likely due to relative size difference between a heparin molecule (molecular weight ∼5000) and a phosphate group (molecular weight ∼80), with Sf9 tau carrying between 1 and 14 phosphates per molecule.^38,71^ Raman spectroscopy has previously been used to measure protein phosphorylation in α-casein indirectly by assessing subsequent changes in protein conformation,^72^ whilst changing environmental pH enabled direct measurement of phosphorylation markers.^39^ As well as phosphorylation, the tau protein undergoes several PTMs that have been linked with disease,^73^ including acetylation,^74^ glycosylation^75^ and others. Raman spectroscopy is sensitive to protein acetylation,^76^ as well as glycosylation^77^ and these measurements are quantifiable.^76,78^ We have demonstrated that the phosphorylation of tau fibrils can be directly detected by Raman spectroscopy. The correlation of phosphorylation and oligomerization in early AD brains,^79^ suggests that the detection of both PTM and conformational state may provide a unique and powerful biomarker for tauopathies. We did not assess the conformation of Sf9 fibrils due to the interference from an associated His-tag. As the impact of phosphorylation is dependent on the phosphorylation pattern,^18^ we have demonstrated that vibrational spectroscopy provides a useful tool to assess tau phosphorylation that may extend to studying the effects of different PTM patterns on tau conformation and aggregation kinetics in future studies.

### The utility of Raman spectroscopy for tauopathy diagnosis

Raman spectroscopy has previously been applied to the diagnosis of tauopathies by the identification of changes in the spectral signature of blood plasma samples.^80–83^ Furthermore, blood serum analysis by FTIR in combination with multivariate analysis has also been used to distinguish AD from healthy controls, as well as from dementia with Lewy bodies (DLB) and FTD.^84,85^ Protein conformers cannot be distinguished in pure serum samples, with spectral differences instead reflecting global changes in response to neurodegenerative disease, such as inflammation. As discussed, protein specific signatures may enable enhanced disease diagnosis and even stratification, yet questions remain whether it is possible to obtain these signatures from biofluids, particularly in blood serum where tau concentrations are extremely low^86^ and the presence of other proteins prevents direct conformational analysis. Strategies to enhance Raman signals such as surface-enhanced Raman spectroscopy (SERS), or the enrichment of tau using antibodies, may enable physiological and pathological concentrations of proteins to be detected, but these techniques come with their own caveats. SERS in particular is not trivial and would require extensive optimization. We have succeeded in optimizing this approach to an extent by showing, in a previous study, its utility for the differentiation of HD patients from controls using blood serum.^87^ It is possible that similar SERS led optimization may enable the detection of tau conformers to enhance the stratification of tauopathies in the future, which may improve the accuracy of disease prognosis. Nevertheless, strategies to obtain Raman signatures of pure proteins from complex mixtures are currently under-developed and require optimization before they can be employed in this manner. A first step in this direction is proof-of-concept study with pure forms of different tau variants studied *in vitro* which we have provided in the current study.

## Experimental

### Purification of 2N4R tau

All buffer reagents are from Sigma unless otherwise stated. 2N4R Tau was purified as reported previously^24^ with some changes. Briefly, pET-29b tau plasmid (addgene, NM_005910) was transfected into *E. coli* BL21 cells for the expression of human tau40 isoform. Bacteria were grown at 37 °C in LB broth with 20 μg mL^−1^ kanamycin until an optical density of 0.5–0.6 was reached at 600 nm absorbance. Expression was induced by adding 1 mM isopropyl β-d-thiogalactopyranoside (IPTG) for 3.5 h. Bacteria were sedimented for 20 min at 5000*g* and stored at −20 °C overnight. Pellets were resuspended in homogenization buffer (20 mM MES, 50 mM NaCl, 1 mM MgCl_2_, 1 mM EGTA, 5 mM DTT, 1 mM PMSF, cOmplete™ Protease Inhibitor Cocktail, pH 6.8) and sonicated on ice. Bacterial cell homogenate was boiled at 95 °C for 20 min followed by centrifugation at 127 000*g* for 45 min at 4 °C. Supernatant was dialyzed against buffer A (20 mM MES, 50 mM NaCl, 1 mM MgCl_2_, 1 mM EGTA, 2 mM DTT, 0.1 mM PMSF pH 6.8) overnight (25 kDa cutoff, Spectra/Por). Samples were then loaded onto a cation exchange column (GE healthcare) and eluted against increasing concentrations of NaCl from buffer B (20 mM MES, 1 M NaCl, 2 mM DTT, 1 mM MgCl_2_, 1 mM EGTA, 0.1 mM PMSF pH 6.8). Fractions were selected and combined based on purity using SDS-PAGE (ESI Fig. S4†). Combined tau fractions were diluted in an excess of ice-cold methanol (1 : 2–1 : 4 volume : volume) and stored overnight at 4 °C for protein precipitation. Protein was sedimented by centrifugation at 4000*g* for 20 min at 4 °C. Methanol was decanted and pellets were dried in a fume hood for 30 min. Pellets were resuspended in a total of 2 mL 8 M guanidine hydrochloride (Gdn HCl, Sigma) and rotated for 1 h at RT to disaggregate any preformed seeds. The buffer was exchanged to PBS (10 mM Na_2_HPO_4_, 2 mM KH_2_PO_4_, 137 mM NaCl, 2.7 mM KCl, 2 mM DTT pH 7.4) or Tris buffer (25 mM Tris buffer, 2 mM DTT, pH 7.0) using a PD-10 desalting column (GE healthcare) as per manufacturer's instructions. Tau protein, p-tau441 (2N4R), was expressed in Sf9 cells and purified as described previously.^71^ Briefly, Sf9 cells were infected with the recombinant virus (pVLhtau40) and incubated for 3 days. A size exclusion column Superdex G200 (GE Healthcare) was used to purify heat-stable tau from heated cell lysate. Protein concentration was measured using absorbance at 280 nm and an extinction coefficient of 7450 cm^−1^ m^−1^. Tau was diluted to 20 μM, snap-frozen in liquid nitrogen, and stored at −80 °C.

### Purification of tau variants tau40, tau23, P301S and K18

Tau variants htau40 WT (2N4R), htau23 WT (0N3R) and 244–372 (K18) were previously cloned into pET-28 as described.^51,54^ P301S was generated using site-directed mutagenesis following the QuikChange protocol from Stratagene/Agilent Technologies. The success of all mutagenesis was confirmed by DNA sequencing. Plasmids containing the desired inserts were first transformed into *Escherichia coli* strain BL21(DE3) and then grown on LB (Miller) agar plates. Single colonies were transferred into LB medium (Miller) and agitated for 15–17 h at 37 °C. The cultures were diluted 1 : 100 with LB medium and again agitated at 37 °C, until optical density reached 0.7–1 at 600 nm. For selection, the growth medium contained kanamycin (50 μg mL^−1^ in agar plates and 20 μg mL^−1^ in solution) (Gold Biotechnology). Protein expression was induced by addition of 0.5 mM isopropyl β-d-1-thiogalactopyranoside (Gold Biotechnology). Cultures were allowed to shake at 37 °C for another 3.5 h before being pelleted at 3000*g* and taken up in resuspension buffer 500 mM NaCl, 20 mM PIPES (Research Products International), pH 6.5, 1 mM EDTA (Fisher Scientific), and 50 mM β-mercaptoethanol (Fisher BioReagents). The cells were heated at 80 °C for 20 min and tip-sonicated (Fisher Scientific sonifier 50% power with a 6 mm tip sonifier) on ice for 1 min before being centrifuged at 15 000*g* for 30 min to separate soluble protein from cellular debris. Soluble Tau was precipitated by gently shaking with 55–60% w/v ammonium sulfate (MP Biomedicals) for 3–20 h at 25 °C. Precipitated Tau pellets from a 15 000*g* spin were taken up in H_2_O with 2 mM DTT (Gold Biotechnology), sonicated for 2 min, syringe-filtered (GxF/GHP 0.45 μm), and loaded onto a cation exchange column (mono S 10/100 GL; GE Healthcare). Proteins were eluted using a linear NaCl gradient (50–1000 mM NaCl, 20 mM PIPES, pH 6.5, 2 mM EDTA), and fractions were pooled based on SDS-PAGE assessment. Pooled ion exchange fractions were loaded onto a Superdex 200 or Superdex 75 (GE Healthcare) gel-filtration column and eluted with 100 mM NaCl, 20 mM Tris (Sigma), pH 7.4, 1 mM EDTA, and 2 mM DTT buffer. Fractions were again assessed using SDS-PAGE and pooled accordingly and then left to precipitate overnight at 4 °C using either an equimolar volume of methanol or a 3-fold volumetric excess of acetone,^54^ along with 5 mM DTT. Following precipitation pellets were collected with a 15 000*g* spin for 10 min, washed with methanol or acetone, and stored at −80 °C until further use.

### Aggregation of 2N4R tau and SF9 tau

For heparin-induced tau aggregation, 20 μM monomeric tau in PBS or Tris buffer (see tau purification section) was combined with low molecular weight heparin (average molecular weight = 5000, Fisher, BP2524) at a 2 : 1 molar ratio, protein : heparin. Tau was then aggregated by incubation at 37 °C in quiescent conditions for 10 days. Fibrils were diluted in PBS or Tris for AFM or were sedimented at 100 000*g* for 45 at 4 °C and resuspended in H_2_O for Raman spectroscopy.

For RNA-induced tau aggregation, 20 μM monomeric tau in PBS was combined with poly(U) (average molecular weight = 100–1000+ kDa, Sigma, P9528) at a 3 : 1 molar ratio (tau : RNA). Tau was then aggregated by incubation at 37 °C in quiescent conditions for 3 days. Fibrils were diluted in PBS for AFM or were sedimented at 100 000*g* for 45 at 4 °C and resuspended in H_2_O for Raman spectroscopy.

### Aggregation of tau variants tau40, tau23, P301S and K18

For tau40, tau23, P301S and K18 tau variants, tau aggregation was previously described as seed preparation and seeded reactions.^51^ In brief, purified 25 μM tau monomers were combined with 50 μM heparin (average molecular weight = 4400, Celsus, EN-3225), 0.5 mM tris(2-carboxyethyl)phosphine (TCEP) (Gold Biotechnology), and buffer 100 mM NaCl, 10 mM HEPES (J. T. Baker) and 0.1 mM NaN_3_ (Fisher Scientific) at pH 7.4, stirring with a Teflon-coated micro stir bar (5 × 2 mm) at 160 rpm for 7–8 days at 37 °C. Fibrils were sedimented at 100 000*g* for 45 at 4 °C and resuspended in H_2_O for Raman spectroscopy.

### Aggregation of β2M

β2M was a kind gift from Eva Scherer. 5 mg mL^−1^ purified β2M in PBS was exchanged into citrate buffer (50 mM citric acid, 100 mM Na_2_HPO_4_, 100 mM NaCl pH 2.5) for long straight fibrils. For short curly fibrils 200 mM NaCl was used. PBS was removed by serial concentration and dilution through a 5 kDa MWCO filter (Vivaspin). β2M was diluted to 1 mg mL^−1^ and incubated at 37 °C with shaking (220 rpm) for 14 days. Fibrils were diluted in citrate buffer for AFM or were sedimented at 16 100*g* for 15 min at 4 °C and resuspended in H_2_O for Raman spectroscopy.

### Raman spectroscopy and sample preparation

A Renishaw InVia microscope system was used for Raman spectroscopy. Quartz coverslips were coated with a hydrophobic surface as described previously.^24^ For drop-deposition Raman spectroscopy (DDRS), 0.5 μL of each protein sample was first dried onto a quartz coverslip under a vacuum and spectra were collected from random locations on the protein spot. The samples were excited using a 785 nm laser focused through a Leica 50× (0.75 NA) short working distance objective for DDRS. Data was obtained and parameters were set using Renishaw WIRE4.1 software. Spectra were collected in the fingerprint region (614–1722 cm^−1^) with an average spectral resolution of 1.09 cm^−1^ (<1 cm^−1^ in amide I region) and cosmic rays were removed after acquisition. The Raman system was calibrated to the 520 cm^−1^ reference peak of silicon prior to each experiment. Erroneous spectra were rejected with unusual background fluorescence that could not be removed using polynomial subtraction.

### Spectral preprocessing and principal component analysis

Preprocessing and Principal Component Analysis (PCA) was performed using the IRootLab plugin (0.15.07.09-v) for MATLAB R2015a.^88^ All spectra were background-subtracted using blank quartz spectra and were smoothed using the wavelet denoising function. A fifth-order polynomial was used to remove fluorescence and the ends of each spectra were anchored to the axis using the rubberband-like function. Spectral intensity normalization was applied using the amide I band or the CH_2_ deformation band. Trained-mean centering was then applied to the spectra before PCA with a maximum of ten principal components.

### Amide I curve-fitting analysis

Amide I curve fitting was performed as reported previously^24^ with some changes. The spectra for each sample were carefully background subtracted using blank/buffer spectra recorded on quartz. The amide I region (1525–1725 cm^−1^) was then truncated using a linear baseline for background subtraction. Second derivative analysis and curve-fitting of the amide I region was performed using mixed Gaussian and Lorentzian on WIRE4.1 software. Four peaks, centered at 1550, 1580, 1606, and 1616 cm^−1^, were assigned to the aromatic amino acids; tryptophan, phenylalanine, and tryptophan (further peaks in this region were added if required to achieve a good fit). Three peaks, representing secondary structure, were centered near 1655 cm^−1^ (α-helix/turns), 1670 cm^−1^ (β-sheet), and 1686 cm^−1^ (nonregular), and a further peak between 1620–1640 cm^−1^ was assigned to nonregular structure/vibrational coupling and was included in secondary structural analysis. The starting curve frequency was determined by comparing the second derivative of the amide I region of all samples and subsequently kept constant for each fitting. The starting curve height was equal to the amide I spectrum at that given frequency. All curves had starting bandwidths at half-height (BWHH) of 15 cm^−1^. Heterogeneous narrowing and broadening of curves was enabled to a maximum of 40 cm^−1^. The percentage of secondary structure was determined by dividing the area under the peak of interest by the sum of the area under each of the peaks used for secondary structural analysis. To avoid computational smoothing of spectra, each fitting was performed on the mean amide I spectrum of each given variant/conformer to achieve suitable signal : noise. No variation was observed when fitting the same amide I spectrum with the same parameters.

### Atomic force microscopy

Tau fibrils were diluted to 2 μM in PBS/Tris and 20 μL was added to a freshly cleaved 10 mm mica disc (Agar Scientific). Protein solutions were incubated at room temperature for 2 min and then washed with 0.22 μM filtered, double distilled H_2_O three times before drying in air. Samples were imaged using a Digital Instruments Multimode IV AFM system operated in tapping mode. Aluminum-coated, noncontact/tapping mode probes with a resonance frequency of 320 kHz and force constant of 42 N m^−1^ were used for all images (Nanoworld, POINTPROBE NHCR). Probes were autotuned using Nanoscope III 5.12r3 software before use. Images were recorded with a scan rate of 1–2 Hz and 512 samples per line/512 lines per image. Images were flattened using WSxM Beta software.^89^

## Conclusions

We have demonstrated the utility of Raman spectroscopy to characterize and distinguish tau conformers, even when these are generated from tau proteins with identical primary sequences. Tau aggregation is complex in disease, as multiple isoforms of tau exist and undergo a wide range of post-translational modifications. Tau protein also interacts with several cofactors and can exist in a range of conformational states, depending on the given disease and potentially disease subtype. We have demonstrated that label-free, spontaneous Raman spectroscopy provides a unique fingerprint that is sensitive to the tau primary sequence, PTM status, cofactor incorporation and conformation in fibrillar aggregates and can report changes therein due to any of these spectral determinants. Importantly, we have provided evidence that the physiochemical properties of the aggregation environment, the associated cofactor and the primary sequence of tau dictate the final fibril conformation. This work sets the benchmark for *in vitro* research related to tau protein, Raman spectroscopy and conformational change including and not limited to; molecular interactions of tau, tau seeding, screening of distinct patterns of PTMs and conformational changes in early/soluble aggregation species, as well as the conformational fingerprinting of tau in different aggregation environments. Raman fingerprints can be used to improve our understanding of tau aggregate polymorphism *in vitro* and may even provide valuable spectral and structural biomarkers in the future.

## Author contributions

Conceptualization: GD, SM, AM. Data curation: GD. Formal analysis: GD. Funding acquisition: BH, MM, SM, AM. Investigation: GD, AC, WR, HW, ZF, CC. Methodology: GD, SM, AM. Project administration: GD, SM, AM. Resources: BH, MM, SM, AM. Validation: GD, SM, AM. Visualization: GD, SM, AM. Writing-original draft: GD. Writing–review and editing: GD, BH, MM, SM, AM.

## Conflicts of interest

There are no conflicts to declare.

## Supplementary Material

RA-011-D1RA00870F-s001
