# Supplementary material for: Conformational fingerprinting of tau variants and strains by Raman spectroscopy
Source: RSC Adv. 2021 Feb 26;11(15):8899–915. doi: 10.1039/d1ra00870f (PMC8330415; doi:10.1039/d1ra00870f)
Supplement: RA-011-D1RA00870F-s001 [file RA-011-D1RA00870F-s001.pdf]

Electronic Supplementary Material (ESI) for RSC Advances.
This journal is © The Royal Society of Chemistry 2021

Supplementary Information

Conformational fingerprinting of tau variants and strains by Raman spectroscopy

George Devitt[1,2,3*], Anna Crisford[1,2], William Rice[2], Hilary A. Weismiller[4], Zhanyun Fan[5], Caitlin Commins[5], Bradley T. Hyman[5], Martin Margittai[4], Sumeet Mahajan[2,3*], Amrit Mudher[1,3*]

[1]School of Biological Sciences, Faculty of Environmental and Life Sciences, University of Southampton, Highfield, Southampton SO17 1BJ, UK.
[2]School of Chemistry, Faculty of Engineering and Physical Sciences, University of Southampton, Highfield, Southampton SO17 1BJ, UK.
[3]Institute for Life Sciences, University of Southampton, Highfield, Southampton SO17 1BJ, UK.
[4]Department of Chemistry and Biochemistry, University of Denver, 2190 E. Iliff Ave., Denver, CO carey80208, USA.
[5]Department of Neurology, Harvard Medical School, MassGeneral Institute for Neurodegenerative Disease, Massachusetts General Hospital, Charlestown, MA, 02129, United States

E-mail: G.T.Devitt@soton.ac.uk, S.Mahajan@soton.ac.uk, A.Mudher@soton.ac.uk

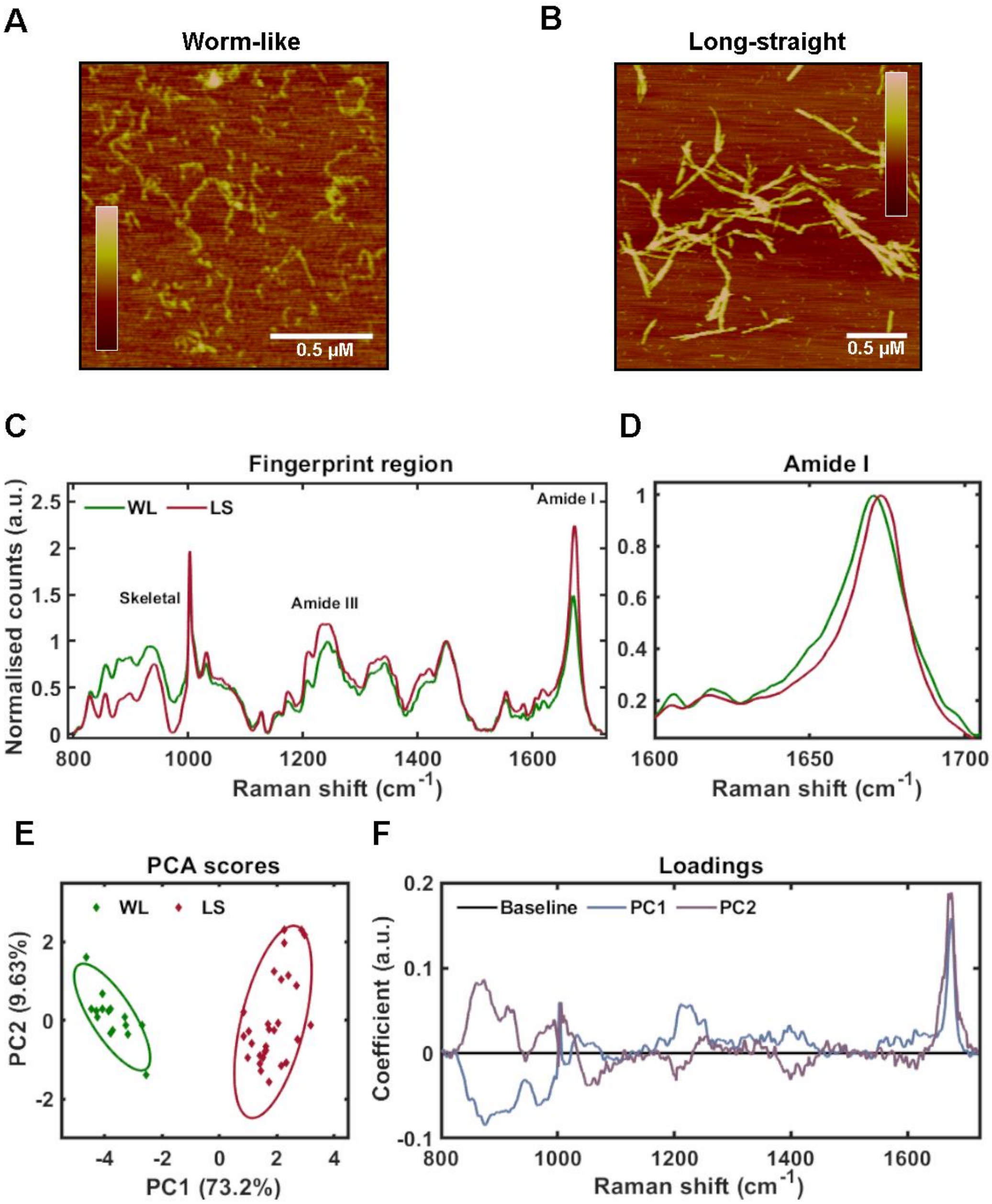

Supplementary Figure 1. Raman fingerprints of β2M fibril strains

A-B) AFM images of β2M fibrils aggregated in high salt buffer (**A,** worm-like) or low salt buffer (**B,** long-straight). Scale bar = 0.5 µm (**A**), 1 µm (**B**), Z scale = 0 nm-15 nm (**A**), 0 nm-25 nm (**B**). **C)** Raman spectra of sedimented β2M fibrils aggregated in in high salt buffer (green trace, WL), or low salt buffer (red trace, LS). Amide I, amide III and skeletal regions are highlighted. **D)** Normalised amide I region for the Raman spectra shown in **C**. **E)** 2-dimensional principal component analysis (PCA) scores plot of Raman spectra shown in **C**. Each solid diamond represents the PC score of a single spectrum. **F**) PC loadings spectra representing the spectral variation responsible for the score across the PC1 axis shown in **C**. Raman spectra represent the class means from multiple spectra; worm-like β2M fibrils: 15, long-straight β2M fibrils: 30.

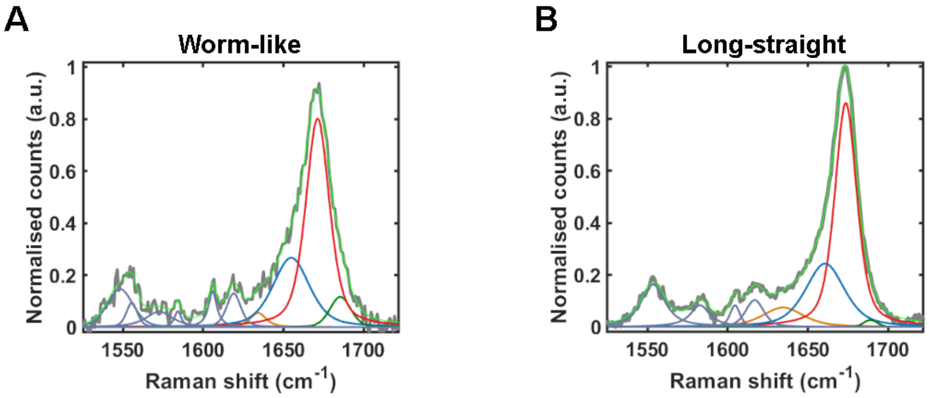

C

Strain	Nonregular/coupling			Turn/α-helix			β-sheet			Nonregular		
	Frequency (cm^{-1})	Width (cm^{-1})	Area (%)	Frequency (cm^{-1})	Width (cm^{-1})	Area (%)	Frequency (cm^{-1})	Width (cm^{-1})	Area (%)	Frequency (cm^{-1})	Width (cm^{-1})	Area (%)
Worm-like	1634	12	3	1655	27	30	1671	18	60	1685	15	7
Long-straight	1634	30	10	1661	29	30	1674	16	60	1689	10	0

Supplementary Figure 2. Secondary structure composition from amide I curve-fitting analysis of β2M fibril strains

Curve-fitting analysis of amide I band (1525-1725 cm^{-1}) from β2M fibril spectra inlcuding; worm-like fibrils (**A**) and long-straight fibrils (**B**). Non-fitted amide I band is shown in grey, with the fitted curve shown in light green. Underlying peaks corresponding to secondary structure are shown in dark green (nonregular), red (β-sheet), blue (turn/helix),and orange (coupling/nonregular). Aromatic amino acid peaks are shown in purple. **C)** Table showing secondary structural composition determined from curve-fitting analysis.

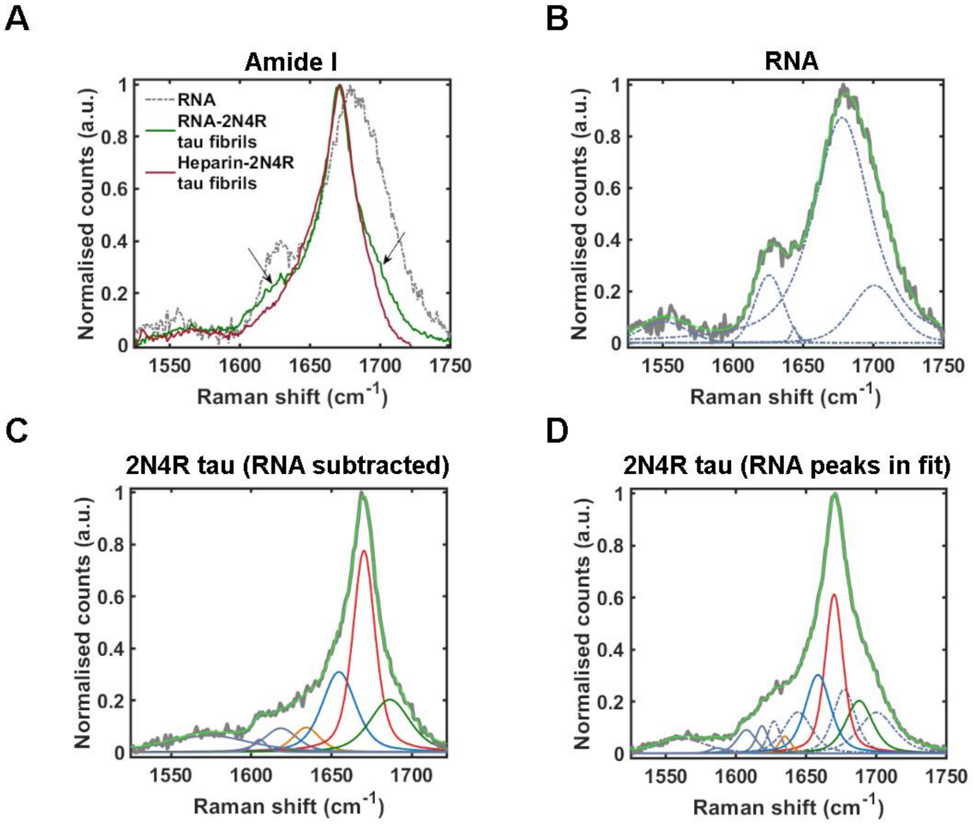

E

	Nonregular/coupling			Turn/α-helix			β-sheet			Nonregular		
Curve-fitting method	Frequency (cm^{-1})	Width (cm^{-1})	Area (%)	Frequency (cm^{-1})	Width (cm^{-1})	Area (%)	Frequency (cm^{-1})	Width (cm^{-1})	Area (%)	Frequency (cm^{-1})	Width (cm^{-1})	Area (%)
RNA spectrum subtracted	1634	20	7	1655	25	27	1670	16	45	1686	30	21
RNA peaks included in fit	1635	10	3	1658	21	31	1670	15	44	1688	23	22
Average	1635	15	5	1657	23	29	1670	16	45	1687	27	21

Supplementary Figure 3. Secondary structure composition from *amide I* curve-fitting analysis of RNA

A) Carbonyl bond stretching/amide I region for neat RNA (grey thatched line), 2N4R tau fibrils generated in PBS using RNA as a cofactor (green line) and 2N4R tau fibrils generated in PBS using heparin as a cofactor (red line). Arrows indicate regions in the amide I spectrum of tau fibrils generated using RNA that align with peaks in the neat RNA spectrum. **B)** Curve-fitting analysis of carbonyl band (1525-1750 cm^{-1}) of the neat RNA spectrum shown in **A** (grey line). **C)** Curve-fitting analysis of amide I band (1525-1725 cm^{-1}) of tau fibril spectra generating using RNA shown in **A** (green line) after 27% subtraction of RNA spectrum shown in **A** (grey line). **D)** Curve-fitting analysis of amide I band (1525-1725 cm^{-1}) of tau fibril spectra generating using RNA shown in **A** (green line) with additional RNA peaks included. The frequency of these peaks were determined from the curve-fit of neat RNA shown in **B**. For curve-fits, the non-fitted amide I band is shown in grey, with the fitted curve shown in light green. Underlying peaks corresponding to secondary structure are shown in dark green (nonregular), red (β-sheet), blue (turn/helix),and orange (coupling/nonregular). Aromatic amino acid peaks are shown in purple. And RNA peaks are shown in thathed-purple. **C)** Table showing secondary structural composition determined from curve-fitting analysis.

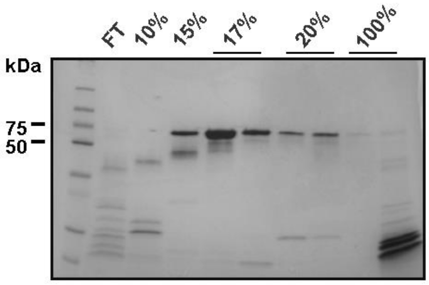

Supplementary Figure 4. SDS-PAGE purity of recombinant tau protein

Eluted fractions from cation exchange chromatography were mixed with Laemmli sample buffer and separated by SDS-PAGE. Protein was visualised using Coomassie Brilliant Blue dye. Full-length 2N4R Tau is found in 15%, 17% and 20% fractions. 17% fractions were typically judged to be the purest and used for subsequent experiments.
